# Plant Poisoning among Children in Rural Sri Lanka

**DOI:** 10.1155/2017/6187487

**Published:** 2017-03-09

**Authors:** M. B. Kavinda Chandimal Dayasiri, Shaluka F. Jayamanne, Chamilka Y. Jayasinghe

**Affiliations:** ^1^University Paediatrics Unit, Lady Ridgeway Hospital for Children, Colombo, Sri Lanka; ^2^Faculty of Medicine, University of Kelaniya, Kelaniya, Sri Lanka

## Abstract

Plant poisoning is a common presentation in paediatric practice and an important cause of preventable mortality and morbidity in Sri Lanka. The burden of plant poisoning is largely underexplored. The current multicenter study based in rural Sri Lanka assessed clinical profiles, poison related factors, clinical management, complications, outcomes, and risk factors associated with plant poisoning in the paediatric age group. Among 325 children, 57% were male with 64% being below five years of age. 99.4% had ingested the poison. Transfer rate was 66.4%. Most had unintentional poisoning. Commonest poison plant was* Jatropha circus* and poisoning event happened mostly in home garden. 29% of parents practiced harmful first-aid practices. 32% of children had delayed presentations to which the commonest reason was lack of parental concern regarding urgency of seeking medical care. Presence of poisonous plants in home garden was the strongest risk factor for plant poisoning. Mortality rate was 1.2% and all cases had Oleander poisoning. The study revealed the value of community awareness regarding risk factors and awareness among healthcare workers regarding the mostly benign nature of plant poisoning in children in view of limiting incidence of plant poisoning and reducing expenditure on patient management.

## 1. Introduction

Acute poisoning in the paediatric age group is an important cause of preventable mortality and morbidity. Globally, every year 3000 children who are less than 14 years of age die following acute poisoning [1]. Children between 1 and 5 years of age have the highest risk for unintentional poisoning [2]. The type of poisons could be one of household poisons, plants, medicines, pesticides, or miscellaneous agents, and plants are identified as an important cause of paediatric poisoning reported to poison information centers [3]. Patterns of plant poisoning vary globally with varying botanical, geographical, and sociocultural characteristics among different populations [4].

Plants remain to be an important cause of mortality among adults in Sri Lanka. Two studies from Northern Sri Lanka reported* Thevetia peruviana* (yellow Oleander) as the commonest plant poison (17% of cases) and it was associated with a case fatality rate of 6-7% [5]. Similar observations were made in subsequent studies from Southern Sri Lanka [6]. Further studies on adult patients from North-Central province showed the unfavorable trend having 32–36% of patients with self-poisoning ingesting* T. peruviana* with a case fatality rate approximating 4% [7]. Studies on plant poisoning involving Sri Lankan children however are limited. A study from Western Sri Lanka [8] identified plants as an important cause of unintentional poisoning in paediatric age group two decades ago. A prospective study (1984–2001) based on predominantly an urban population in Sri Lanka reported that plants accounted for 10% of poisoning events with a case fatality rate of 0.8% [9]. Up to date there has been no evidence from either prospective or retrospective studies with regard to plant poisoning among children from rural Sri Lanka. This multicenter study has described the patterns of plant poisoning in North-Central province of Sri Lanka (NCP) which accommodates a predominant rural community.

## 2. Methods

This multicenter prospective study included 36 hospitals in the North-Central province of Sri Lanka (Anuradhapura Teaching hospital (THA), Polonnaruwa District General hospital (THP), and 34 base/district/rural hospitals of the province under RDHS, Regional Director of Health Services). The study was conducted in four major arms: (1) a two-year prospective study (2012–2014) at Anuradhapura Teaching Hospital, (2) a two-year prospective study (2012–2014) at Polonnaruwa general hospital, (3) one-year prospective study involving 34 hospitals under RDHS of NCP (2013-2014), and (4) a five-year retrospective study at Anuradhapura Teaching Hospital (2007–2012). The major part of data collections was carried out at Anuradhapura hospital prospectively by the same investigator using a pretested, multistructured, interviewer administered questionnaire that comprehensively assessed clinical profiles, plant poison related factors, clinical management, complications, and outcome following acute paediatric poisoning.

Both children with intentional and unintentional poisoning were included in the assessment. Children aged 9 months to 12 years were considered for the analysis. Acute poisoning due to nonplant poisons (household poisons/medicines/pesticides), food poisons, snake envenomation, allergic reactions, and adverse drug reactions which can be considered in the purview of toxicology was omitted in the study. Also children with doubtful poisoning where there was no clear aetiology were excluded from the study. All three prospective studies identified clinical presentations, reasons for delayed management in addition to demographic data.

A prospective controlled risk factor study included all children who presented with plant poisoning to Anuradhapura Teaching Hospital over the two-year study period (2012–2014). The controls were selected from the same hospital and children who presented with acute medical illnesses were recruited as controls. The acute medical illnesses considered included viral fever, acute upper respiratory tract infection, and urticaria. All other acute conditions including nonspecific symptoms without a definitive diagnosis were excluded. All children were matched for age and gender on a case by case basis.

Data collections in all components of the current study were subjected to independent audit and close monitoring by South Asian Clinical Toxicology Research Collaboration (SACTRC) and the investigators of the study themselves. Ethical clearance for the study was issued by ethical review committees, faculty of medicine, University of Kelaniya, and Rajarata University of Sri Lanka.

## 3. Results

There were 325 incidents of plant poisoning reported in all arms of the study. Male children outnumbered female children in all studies and amounted to 186 (57%). Sixty-seven percent of children were less than five years of age. However, plant poisoning was uncommon among children who were less than two years of age (4.6% of total) compared to older children. The majority of poisoning events were secondary to unintentional ingestion of the poison (314/325, 96.6%). Mortality rate was 1.2% (4 cases) and all four cases (100%) followed ingestion of lethal dose of Oleander. Sixty-six percent of children (216/325) were transferred from a local hospital (under RDHS) to a tertiary care hospital following the poisoning event.  Table 1 has compared the demographic characteristics, patterns of poisoning, and transfer rates of children in different arms of the study.

*Jatropha curcas* (Barbados nut/physic nut/poison nut) was the commonest plant poison ingested by children and it was consistently seen in all studies. Poisoning with* Thevetia peruviana* (Oleander) and* Abrus precatorius *(rosary pea/paternoster pea/wild licorice) was commonly seen after* Jatropha curcas*. Poisoning with those three plants comprised 83.4% of total plant poisonings. Patterns of plant poisoning were similar and consistent in all studies.  Table 2 has presented all children with poisoning based on the plant ingested.

Commonest route of poisoning was ingestion (323/325, 99.4%). One child had symptoms following contact of the poison with mucus membranes. Another child indirectly ingested the poison indirectly through breast milk following her mother intentionally ingesting Oleander.

### 3.1. Comparison of Clinical Manifestations and Reasons for Delayed Presentations to Primary Care Hospital

160 children recruited to studies at THA, THP, and RDHS were available for the analysis. Gastrointestinal symptoms (125 children, 78.1%) were the predominant symptoms following plant toxin ingestion and they were consistently seen in all three studies. Most gastrointestinal symptoms occurred following* Jatropha circus* intoxication. All children with cardiovascular symptoms were intoxicated with Oleander.  Table 3 illustrates the variability in clinical manifestations in detail.

Fifty-two children (32.5%) presented to primary care hospital at least two hours after the ingestion of the poison. Commonest reason for delayed presentation was lack of concern regarding urgency of the situation, 33 children (20.6%). Detailed analysis of reasons for delayed presentation to primary care unit is presented in Table 4.

### 3.2. Detailed Evaluation of Patterns and Risk Factors of Plant Poisoning among Children at THA

Sixty-five children presented to THA following plant poisoning over the two-year study period. Mean age of children was 4.3 years (range: 11 months–12 years). Most parents had received secondary education, 50 fathers (77%) and 54 mothers (83.1%). The majority of fathers were engaged in farming (21, 32.3%), defense service (11, 16.9%), and manual labour (11, 16.9%). Most mothers were housewives (45, 69.2%). Most of the poisoning events occurred in home garden (43, 66.1%) followed by cultivation area (12, 18.4%) and inside of home (5, 7.7%).

Harmful first-aid measures were practiced in 19 children (29.2%). The commonest measure was forceful ingestion of water (8, 12.3%) and it was followed by forceful finger insertion (3, 4.6%), forceful milk (2, 3.1%), and coconut milk ingestion (2, 3.1%).

The majority of children had onset of symptoms within one hour from the time of poisoning event (35, 53.8%). Though most children (45, 69.2%) were brought to primary care unit within two hours from the poisoning event, twenty children (31.8%) presented at least 2 hours after the poison was ingested (range: 2 to 8 hours). Emesis induction was offered to 32 children (49.2%). Two children (3.1%) required prescription of antidotes and management in an intensive care unit. Reported medical complications included cardiac arrhythmia (2) (3.1%), severe dehydration (1) (1.5%), hematemesis (1) (1.5%), and seizures (1) (1.5%).

Risk factor evaluation showed four proposed risk factors which were associated with significantly elevated risk (*p* < 0.001) of plant poisoning among children. They were the presence of poisoning pants in home garden, inadequate supervision of the child, past history of poisoning, and parents' subjective feeling of lack of family support to look after children. Five proposed risk factors did not reveal a significant association with plant poisoning (working mother, children with deprived schooling, young mother, poorly educated mother, farming parents, and parents' subjective perception of having economic problems).  Table 5 has compared the presence of proposed risk factors in the two groups.

## 4. Discussion

Plant poisoning in children is one of the common presentations to emergency departments. However, the risk factors and circumstances of these poisoning events are largely underexplored in international literature [10] and most studies have concentrated on data reported to poison information centers. The pattern of plant poisoning varies considerably based on age, sex, and sociodemographic factors among different geographic regions [11]. Young children comprise a unique group in that they have the natural curiosity about their surroundings and tendency for oral exploration keeping them highly vulnerable to unintentional poisoning [12].

The current study identified male children more vulnerable than female children for plant poisoning. Furthermore, it also revealed that the majority of children were between 2 and 5 years of age. Unintentional poisoning accounted for more than 90% of poisoning events and almost all cases had taken the poison through ingestion. Similar observations are made in previously published studies from Sri Lanka [9], India [10], Thailand [13], and Taiwan [14].

A key observation in the current study was the higher transfer rate of children. The study identified that 66.4% of total population were transferred between two hospitals and 67.2% of children who attended local hospitals under RDHS were subsequently transferred to a tertiary hospital for further management. Previously published adult studies in the same region of Sri Lanka observed a transfer rate of 50% [15] which is lower than current figures. Despite higher transfer rates, case fatality and complication rates in current study were significantly lower than adult studies [7]. Transferring of patients significantly adds to healthcare expenditure and the average patient cost per transfer was US$ 14.03 in one study eight years before in the same region [16]. These facts reveal the value of increased awareness among healthcare workers in rural territories regarding the nature and outcome of plant poisoning in the paediatric age group which is mostly benign. Effective triage and limitation of transfers only to needy children would likely cut down healthcare expenditure, duration of hospital stay, and effect on families of children with plant poisoning.

We observed a higher percentage of deliberate ingestions compared to a previously published study in urban Sri Lanka [9]. In that study, the common plant poisons were* Jatropha circus, Ricinus communis, *and* Jatropha multifida *(physic nut/nettle spurge) accounting for 43% of total plant poisonings. In the current study based on NCP,* Jatropha circus*, Oleander, and* Abrus precatorius* were identified in 83.4% of all plant poisonings. All deliberate poisonings happened by ingestion of Oleander. These poisonous plants were usually found in fences of houses, by sides of walking paths and canals. Some were in fact grown as house plants by parents. Higher proportion of deliberate poisoning with Oleander raises the question as to how they learn these behaviours given the fact that NCP has higher numbers of suicides among adults [1].

It was also revealed that 78.5% of children had ingested plant poisons within their own home gardens or neighborhoods. This figure of 78.5% is much higher compared to previous Sri Lankan studies conducted in more urban areas [9] and underpins hazardous nature of home gardens and neighborhoods of remote villages which accommodate poisonous plants without being noticed by house owners. Higher rates of transfers from many rural territories within the province compared to other poison types [17] also highlight widespread distribution of environmental risk factors within the community.

Home garden was the commonest place for poisoning with plant seeds in current study. A study from urban Sri Lanka observed* Alocasia* (elephant's ear plant) as the commonly encountered plant poison in home garden [18]. In our study, commonly encountered plant poisons in home gardens were* Jatropha circus, Abrus precatorius,* and Oleander. Home gardens in remote areas of NCP are less cleared allowing growth of these plants and most parents were unaware of the potential toxicity.* Jatropha circus* was identified as the commonest poison in current study and* Jatropha circus* induced gastrointestinal symptoms were the commonest clinical presentation. The same observation has been made in studies from other parts of Sri Lanka [9] and Asia [13].

Patterns of poisoning and as well as subsequent outcome are always related to the underlying sociocultural circumstances. Observation of scientifically unproven yet culturally based first-aid practices by some parents as observed in this study can be associated with detrimental effects. Therefore providing knowledge to at-risk communities regarding such issues is helpful in bringing down childhood poisoning related morbidity and mortality.

Delayed presentation to primary care hospital following the poisoning event has a potentially strong negative impact on effective management and patient outcomes [19]. In the current study, the commonest reason for delayed presentation had been lack of concern regarding the urgency for seeking medical care. Child remaining asymptomatic after ingestion, lack of identity of the ingested substance as a poison, and small ingested amount of poison had kept a higher threshold for some parents to seek urgent medical attention. The duration of hospital stay and severity of complications have been shown to have a direct correlation with lag time in reaching the hospital following the poisoning event [20]. These facts reveal the value of community awareness in seeking early primary care in improving patient outcomes.

The current study identified four factors including presence of poisoning plants in home garden, inadequate supervision, past history of poisoning, and parents' subjective feeling of lack of family support to look after children as being associated specifically with significantly elevated risk of plant poisoning in children. Schmertmann et al. reported inadequate supervision as a risk factor for unintentional poisoning in the paediatric age group [21]. Similarly, past history of poisoning has been identified as a risk factor for acute poisoning [22]. However, evidence regarding risk factors for plant poisoning by properly controlled studies is scant in currently available literature and further studies are needed. As majority of plant poisoning occurred within home premises in the current study, a holistic approach which targets home environment would help in managing the burden of poisoning.

## 5. Conclusion

The study revealed harmful effects of traditional first-aid practices which are detrimental to health of the child. Children become victims of plant poisoning mostly secondary to presence of poisoning plants in home garden, inadequate supervision by care givers, and previous poisoning. As these risk factors are significantly associated with plant poisoning, the effect of community education to enhance vigilance and assurance of safe environment should be evaluated. The study also identified the importance of awareness among healthcare workers regarding the mostly benign nature of plant poisoning in children in view of reducing expenditure on patient management.

## Figures and Tables

**Table 1:** Demographic characteristics, patterns of poisoning, and transfer rates of children with plant poisoning.

Variable	Retrospective study (*n* = 165)	THA Study (*n* = 65)	Polonnaruwa study (*n* = 58)	Peripheral study (*n* = 37)	Total (*n* = 325)
(1) Male : female	90 : 59 4.5% : 45.5%	38 : 27 58.4% : 1.6%	38 : 20 65.5% : 34.5%	20 : 17 54% : 46%	186 : 139 57% : 43%
(2) <5 years : >5 years	99 : 66 60% : 40%	52 : 13 80% : 20%	26 : 32 44.8% : 55.2%	31 : 6 84.8% : 15.2%	208 : 117 64% : 36%
(3) Unintentional : intentional	162 : 3 98% : 2%	60 : 5 92% : 8%	57 : 1 98.3% : 1.7%	35 : 2 95.6% : 5.4%	314 : 11 96.6% : 3.4%
(4) Mortality	2 (1.2%)	1 (1.5%)	1 (1.7%)	—	4 (1.2%)
(5) Commonest poison	*Jatropha circus*	*Jatropha circus*	*Jatropha circus*	*Jatropha circus*	*Jatropha circus*
(6) Transfer rate	108 (65.4%)	49 (75.3%)	34 (58.2%)	25 (67.6%)	216 (66.4%)

**Table 2:** Patterns of poisoning with poisonous plants among children in North-Central province.

Poisonous Plant	Retrospective study (*n* = 165)	Prospective study (*n* = 65)	Polonnaruwa study (*n* = 58)	Peripheral study (*n* = 37)	Total (*n* = 325)
(1)* Jatropha curcas*	81 (49.1%)	22 (33.8%)	24 (41.3%)	16 (43.2 %)	143 (44%)
(2)* Thevetia peruviana*	36 (21.8%)	10 (15.4%)	14 (24.1%)	8 (21.6%)	68 (20.9%)
(3)* Abrus precatorius*	27 (16.4%)	15 (23%)	11 (19%)	7 (18.9%)	60 (18.5%)
(4)* Ricinus communis*	9 (5.4%)	5 (7.7%)	1 (1.7%)	2 (5.4%)	17 (5.2%)
(5)* Jatropha multifida *	6 (3.6%)	4 (6.2%)	2 (3.3%)	— (0%)	12 (3.7%)
(6)* Caladium*	1 (0.6%)	3 (4.6%)	2 (3.3%)	2 (5.4%)	8 (2.5%)
(7)* Datura stramonium *	1 (0.6%)	2 (3.1 %)	1 (1.7%)	1 (2.7%)	5 (1.5%)
(8)* Gloriosa superba*	1 (0.6%)	2 (3.1%)	1 (1.7%)	— (0%)	4 (1.2%)
(9)* Other*	3 (1.8%)	2 (3.1%)	2 (3.3%)	1 (2.7%)	8 (2.4 %)

**Table 3:** Clinical manifestations of plant poisoning among children in rural Sri Lanka.

Reasons for delayed presentation	THA	THP	RDHS	Total
(1) Gastrointestinal symptoms	59 (90.1%)	41 (70.6%)	25 (67.5%)	125 (78.1%)
(2) Respiratory symptoms	25 (38.4%)	22 (37.9%)	16 (43.2%)	63 (39.3%)
(3) Cardiovascular symptoms	3 (4.6%)	6 (10.3%)	2 (6.4%)	11 (6.8%)
(4) Neurological symptoms	2 (3%)	1 (1.7%)	—	3 (1.8%)

**Table 4:** Reasons for delayed presentation to primary care unit among children in rural Sri Lanka.

Reasons for delayed presentation	THA	THP	RDHS	Total
(1) Lack of concern regarding need for urgent care	12 (18.4%)	11 (18.9%)	10 (27%)	33 (20.6%)
(2) Lack of transport facilities for emergency management	8 (12.3%)	7 (12%)	12 (32.4%)	25 (15.6%)
(3) Lack of knowledge regarding possible complications	6 (9.2%)	6 (10.3%)	4 (10.8%)	16 (10%)
(4) Lack of financial resources	6 (9.2%)	4 (6.8%)	2 (5.4%)	12 (7.5%)
(5) Poisoning event unrevealed until symptoms occurred	2 (3.1%)	1 (1.7%)	1 (2.7%)	4 (2.5%)
(6) Delayed attention by medical team	1 (1.5%)	—	—	1 (0.6%)

**Table 5:** Analysis of proposed risk factors in case-control study.

Proposed risk factor	Cases	Controls	Chi square value	*p* value
(1) Poisonous plants in home garden	54	6	71.31	<0.001
(2) Inadequate supervision of the child	49	13	39.96	<0.001
(3) Past history of poisoning	24	2	23.26	<0.001
(4) Lack of family support	33	12	14.98	<0.001
(5) Mother working during the daytime	17	15	0.16	0.68
(6) Lack of schooling/education in mother	2	1	0.34	0.55
(7) Young mother (<19 years)	7	9	0.28	0.59
(8) Primary level education in mother	10	7	0.60	0.43
(9) Parents from farming community	22	19	0.32	0.57
(10) Economic problems	21	20	0.03	0.85
